# Painless Excavation by Anæsthetizing the Tooth with Ether Spray

**Published:** 1888-07

**Authors:** 


ARTICLE VII.

### PAINLESS EXCAVATION BY ANÆSTHETIZING THE TOOTH WITH ETHER SPRAY.

BY DR. OTTOLENGUI.

Isolate the tooth and one or two others on each side of it, with rubber dam. After using bibulous paper, apply dry heat, not too hot, with a chip blower. These blasts should be continued until the whole tooth becomes whitened, which is sufficient evidence of dehydration.

Having done this, the next step is to anæsthetize the fiber. To do this use the purest ether, thrown on with a continuous spray apparatus. An atomizer with double bulbs and various tips is best. The length of time that this should be continued may be known by watching the patient's eyes. The ether will cause always more or less pain, therefore, the patient should be warned of this in advance; it being also explained that the pain will be a decreasing one, and that the result will be that the tooth may be prepared without pain. Then it will be found that the patient thus prepared will endure the pain, but will demonstrate his suffering by contracting the eyebrows; a gradual relaxation of those parts will ensue till a placid, indifferent expression will present, when it may be understood that all pain has ceased; the spray may be continued a little beyond this and then the tooth operated on. The result of this method is invariable. Wherever the conditions are complied with success is inevitable. There is no exception to this statement. The patient and operator are both delighted always; the former being saved pain, and the latter time, and if of a sympathetic nature, nervous exhaustion.

A few words of caution: The ether, if allowed to come in contact either with the mucous membrane in the mouth or with the cheek itself, is very unpleasant and often painful. To avoid the former a large piece of dam is required and each tooth should be securely tied. As to the latter, when either of the superior front teeth are to be operated on, a towel should be placed around the face and over the chin, passing up under and near to the tooth, and then held in place by a rubber dam holder, an elastic which passes behind the head, with a clamp at each end. This is so arranged that as the ether is sprayed it runs down towards and is taken up by the towel In other cases less accessible a piece of spunk must be placed near the tooth which will act similarly.

It has been objected that such treatment endangers the life of the pulp. Experiments have been made, the spray being pointed at the pulp itself, exposed, and such pulp has been saved alive afterwards. Another objection raised was that with the tooth so thoroughly under control as this process allows, there is a temptation to insert a filling which may give great trouble when the tooth returns to normal sensibility. This is answered readily. Such teeth must be filled, and whatever it is safe to fill them with without controling the sensitiveness, will be equally safe with the tooth anæsthetized. If the cavity is deep, a capping should cover the dentine before inserting gold.

After treating a tooth in this way, the operator is enabled to use cutting instruments at will for from two to five minutes, without causing the least pain. The method is better adapted to the upper teeth for the reason that the ether accumulating in the cavity of a lower tooth, prevents the full force of the spray being transmitted. Nevertheless if a piece of spunk be properly arranged to take off the excess the result will be as good in the inferior as in the superior jaw.

There is little left to say beyond the statement that if this method is only tried it will be adopted. A boon to both operator and patient is here offered, and it seems surprising how slow the profession are to even examine into the claims made. It is to be hoped that before many months may dentists besides the writer may be giving their patients the benefit of this immunity from the pangs of sensitive dentine.—*Archives of Dentistry.*

